# Supplementary material for: Collective nuclear behavior shapes bilateral nuclear symmetry for subsequent left-right asymmetric morphogenesis in Drosophila
Source: Development. 2021 Apr 26;148(18):dev198507. doi: 10.1242/dev.198507 (PMC8126412; doi:10.1242/dev.198507)
Supplement: Supplementary information [file develop-148-198507-s1.pdf]

Script 1. measures the average distance between nuclei and the midline.

```
# Usage - select nuclei and execute this script

import maya.cmds as cmds
import math as math
import numpy as np

std_list = []
to_midline = []
cal_by_curve = []

print "\n>>>start<<<\n"
width_ = 150
# now width of embryo is 150
sel_ = cmds.ls(sl=1)

for serial in sel_:
        XYZ = cmds.objectCenter(serial, gl=True)
        # getting the center of nucleus
        middd = [0, 0, 0]
        list_XZ = XYZ[0], XYZ[2]

        dx = 0 - XYZ[0]
        dz = 0 - XYZ[2]

        dis_to_cent = math.sqrt((dx*dx)+(dz*dz))

        print "nucleus =", serial
        print "real distance to midline =", dis_to_cent

        middd[1] = XYZ[1]
```

1/2

```
        to_midline.append(XYZ)
        to_midline.append(middd)

        print "coordinate of nucleus and midline =", to_midline

        cc = cmds.curve(d=1,p=to_midline)
        print "recheck length by line", (cmds.arclen(cc))
        ratio_ = (dis_to_cent / width_) * 100
        print "distance as ratio =", ratio_
        std_list.append(ratio_)

        ratio_by_curve = ((cmds.arclen(cc) / width_)) * 100
        cal_by_curve.append(ratio_by_curve)

        del to_midline[:]

mean_ = np.mean(std_list)  #calculate mean
std_ = np.std(std_list)    #calculate standard deviation
mean_curve = np.mean(cal_by_curve)  #calculate mean by line
std_curve  = np.std(cal_by_curve)   #calculate standard deviation by line

print "\n<result>\n"
print "width of embryo =\n", width_
print "number of connections =\n", len(sel_)
print "mean of connectiones =\n", mean_
print "standard deviation =\n", std_
print "mean of connectiones (by line) =\n", mean_curve
print "standard deviation (by line) =\n", std_curve
```

2/2

Script 2. calculates the collectivity of nuclear arrangement.

```
# Usage - select nuclei and execute this script

import maya.cmds as cmds
import numpy as np

print "\n>>>start<<<\n"
width_ = 107.94
#now width of embryo is 107.94

sel_ = cmds.ls(sl=1)

pos_ = []
curves_ = []
for serial in sel_:
			XYZ = cmds.objectCenter(serial, gl=True)
			#calculate center of nucleus
			print "coordinate of", serial, XYZ
			pos_.append(XYZ)

sort = sorted(pos_, key=lambda pos_: pos_[1])
#in case of ‘pos_[1]’, nearest nuclei are connect along Y axis
#if case of ‘pos_[0]’, nuclei are connect along X axis
#and ‘pos_[2]’, nearest nuclei are connect along Z axis
```

1/2

```
print "sorted\n", sort
cc = cmds.curve(d=1,p=sort)
print "length of total distance = "
print cmds.arclen(cc)
print "number of connections = "
ii = (len(sort)) - 1
print ii
for i in range(0,len(sort)):
    if i < ii:
        a = np.array(sort[i])
        b = np.array(sort[i+1])
        dist = np.linalg.norm(a-b)
        #real distance
        ratio__ = (dist / width__) * 100
        #real distance is changed as ratio
        print "nucleus #", i
        print "real distance =", dist
        print "ratio =", ratio__
        curves_.append(ratio__)
    else:
        print "\n<result>\n"
print "length of each distances=\n", curves_
mean_ = np.mean(curves_)  #calculate mean
std_ = np.std(curves_)    #calculate standard deviation
print "width of embryo =\n", width_
print "number of connections =\n", len(sel_)
print "mean of connectiones =\n", mean_
print "standard deviation =\n", std_
```

2/2

Graph 1. Number of nuclei

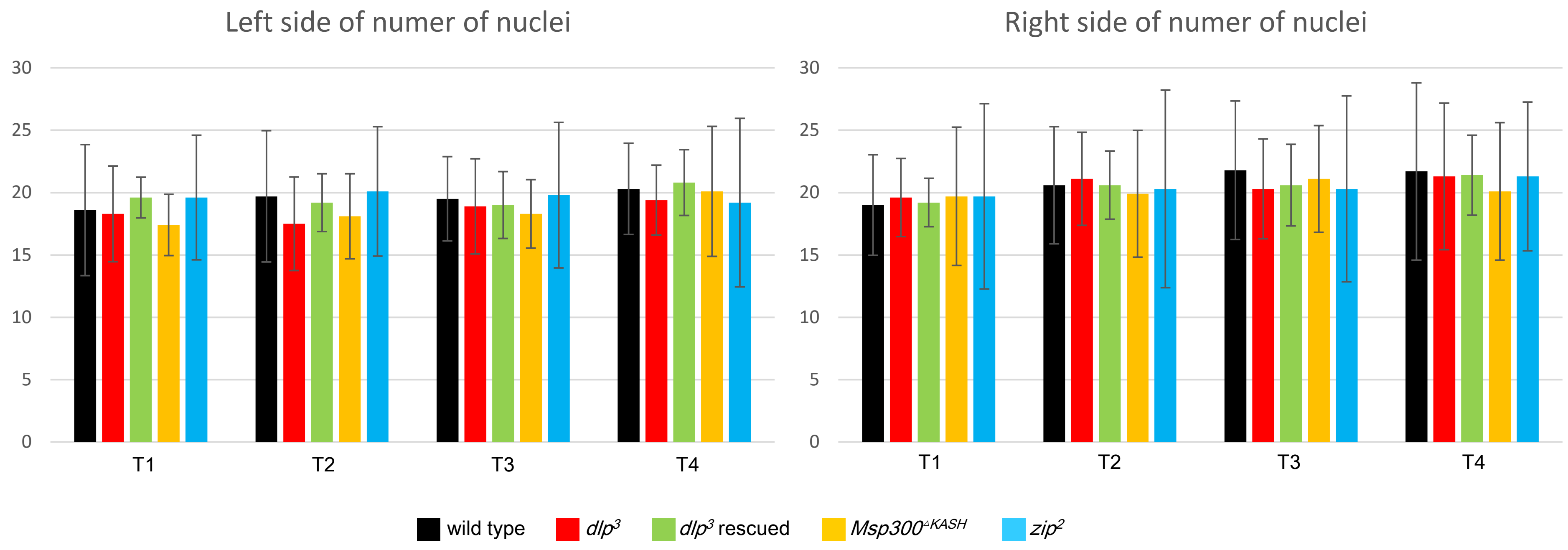

Development • Supplementary information

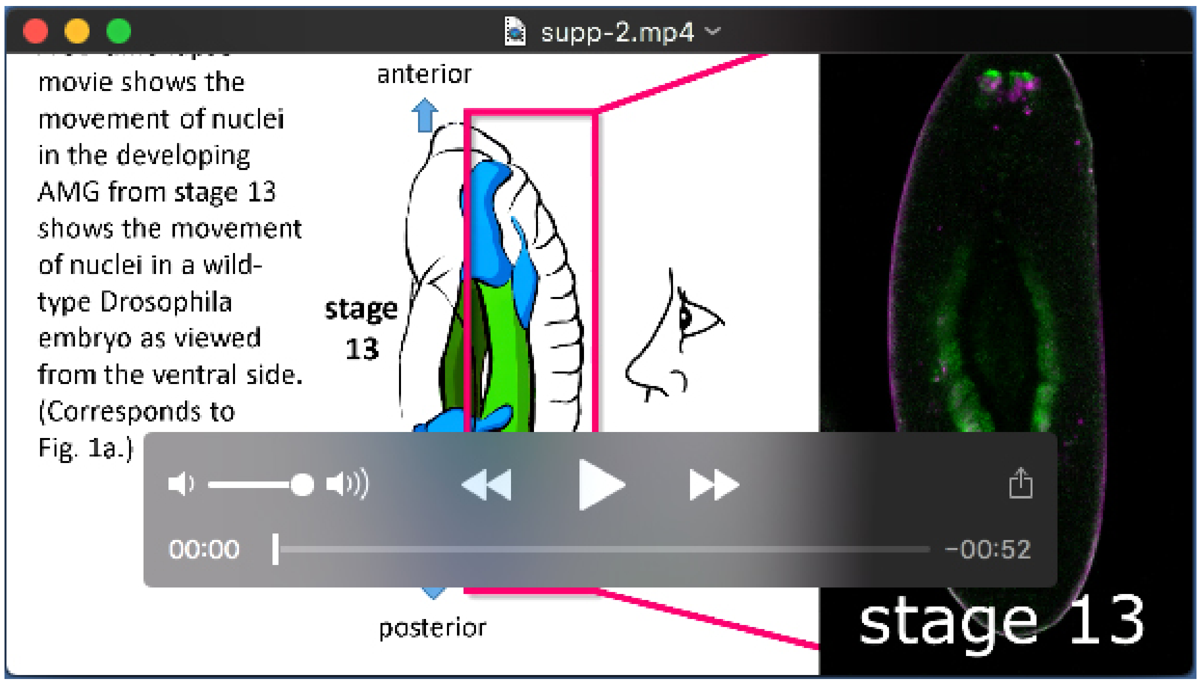

Movie 1

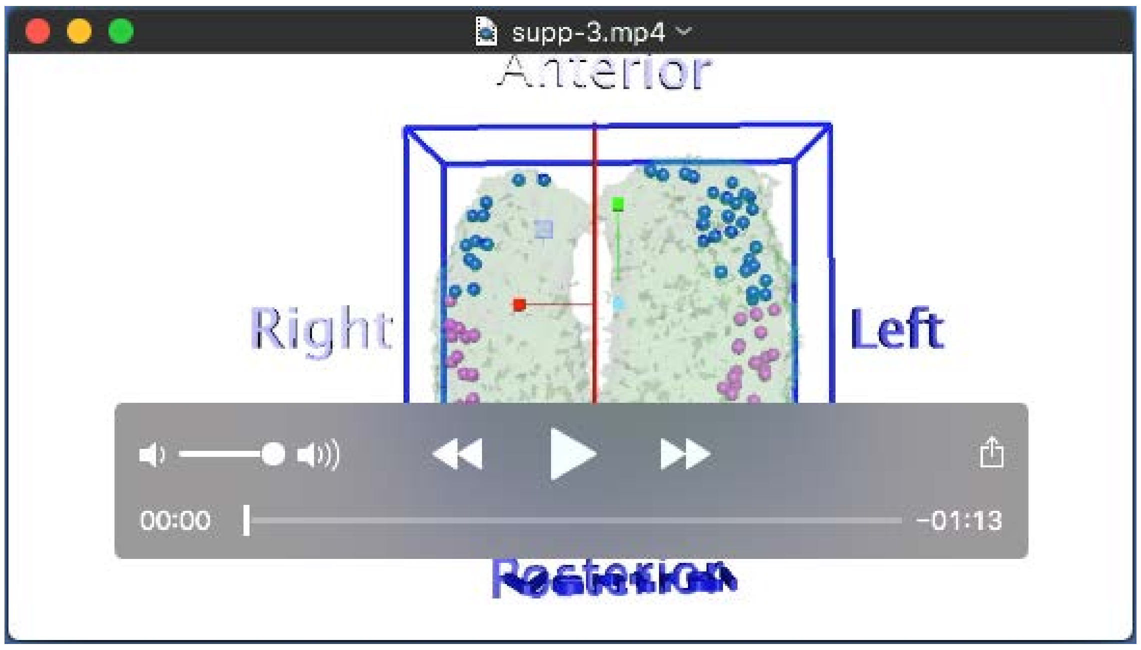

Movie 2

Development • Supplementary information

Fig. S1

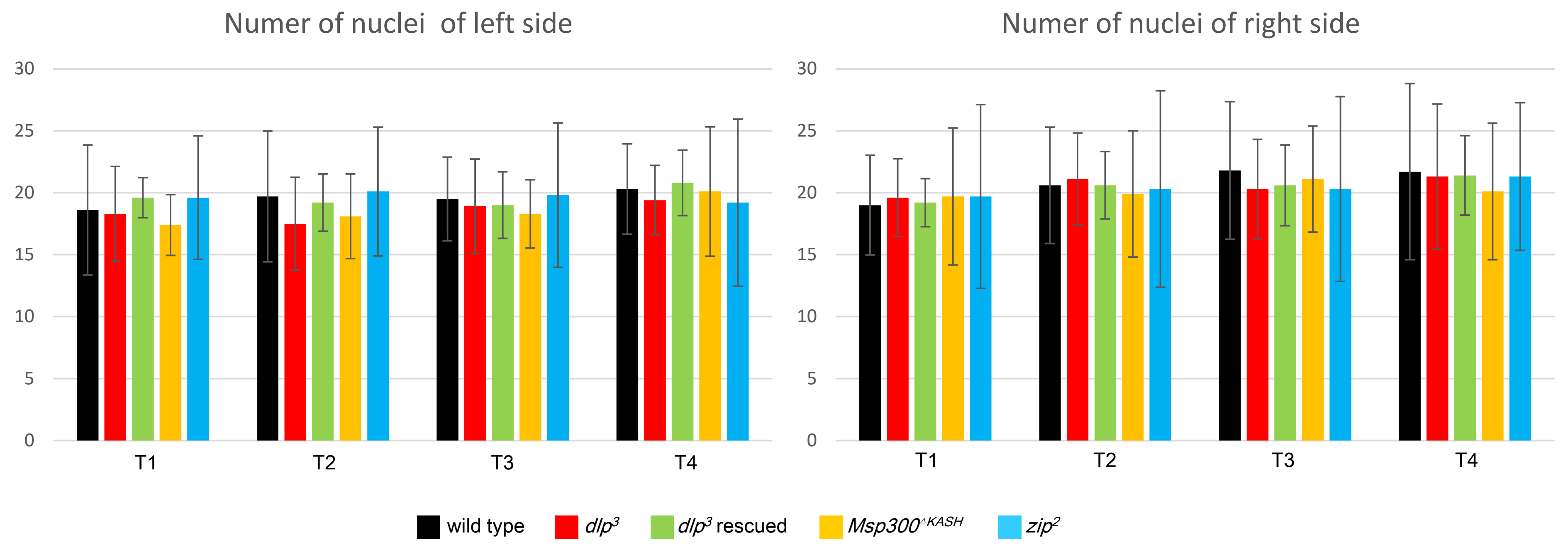

Fig. S1. The number of nuclei examined at stage T1-T4 is shown. The average number of nuclei in each embryo was 20.1±4.8

Development • Supplementary information
